# Biodegradation of Microcystins by Aquatic Bacteria *Klebsiella* spp. Isolated from Lake Kasumigaura

**DOI:** 10.3390/toxins17070346

**Published:** 2025-07-10

**Authors:** Thida Lin, Kazuya Shimizu, Tianxiao Liu, Qintong Li, Motoo Utsumi

**Affiliations:** 1 Graduate School of Science and Technology, University of Tsukuba, 1-1-1 Tennodai, Tsukuba 305-8572, Japan; s2036029@u.tsukuba.ac.jp (T.L.); ltx-mail@163.com (T.L.); 2 Faculty of Life Sciences, Toyo University, 1-1-1 Izumino, Ora-gun, Itakura 374-0193, Japan; k_shimizu@toyo.jp; 3 Faculty of Life and Environmental Sciences, University of Tsukuba, 1-1-1 Tennodai, Tsukuba 305-8572, Japan; 4 College of Engineering, Shibaura Institute of Technology, Tokyo 135-8548, Japan; lqtli@shibaura-it.ac.jp; 5 Microbiology Research Center for Sustainability, University of Tsukuba, 1-1-1 Tennodai, Tsukuba 305-8572, Japan

**Keywords:** microcystin, biodegradation, *Klebsiella*, *mlrA* gene, Lake Kasumigaura

## Abstract

Microcystins (MCs) are the most toxic and abundant cyanotoxins found in natural waters during harmful cyanobacterial blooms. These toxins pose a significant threat to plant, animal, and human health due to their toxicity. Degradation of MCs by MC-degrading bacteria is a promising method for controlling these toxins, demonstrating safety, high efficiency, and cost-effectiveness. In this study, we isolated potential MC-degrading bacteria (strains TA13, TA14, and TA19) from Lake Kasumigaura in Japan and found that they possess a high capacity for MC degradation. Based on 16S rRNA gene sequencing, all three isolated strains were identified as belonging to the *Klebsiella* species. These bacteria effectively degraded MC-RR, MC-YR, and MC-LR under various temperature and pH conditions within 10 h, with the highest degrading activity and degradation rate observed at 40 °C. Furthermore, the isolated strains efficiently degraded MCs not only under neutral pH conditions, but also in alkaline environments. Additionally, we detected the MC-degrading gene (*mlrA*) in all three isolated strains, marking the first report of the *mlrA* gene in *Klebsiella* species. The copy number of the *mlrA* gene in the strains increased after exposure to MCs. These findings indicate that strains TA13, TA14, and TA19 significantly contribute of MC bioremediation in Lake Kasumigaura during cyanobacterial blooms.

## 1. Introduction

Harmful cyanobacterial blooms (HCBs) are a significant issue in eutrophicated lakes, ponds, and reservoirs, especially during the summer season, worldwide. About 70% of cyanobacterial blooms produce the cyanotoxin: microcystin (MC), which is widely distributed and the most detected among the cyanotoxins worldwide [1]. MCs are one of the most hazardous groups of cyanotoxins generated by HCBs. They consist of cyclic heptapeptide structures made up of seven amino acids and are produced by cyanobacteria from genera such as *Microcystis*, *Anabaena*, *Cylindrospermopsis*, *Nostoc*, and *Planktothrix* [2,3]. The chemical structure of MCs is cyclo-(D-Ala-X-D-MeAsp-Z-Adda-DGlu-Mdha), where X and Z are variable L-amino acids. Over 270 variants of MCs have been identified, including microcystin-LR (MC-LR), microcystin-RR (MC-RR), microcystin-YR (MC-YR), and so on [4,5,6,7]. MCs are extremely stable, highly soluble in water, non-volatile, and resistant to heat and hydrolysis due to their cyclic structure [8,9,10].

The degradation of each MC variant by isolated bacteria has been investigated. For instance, *Sphingomonas* sp. NV3, *Sphingopyxis* sp. a7, USTB-05, m6, *Klebsiella* sp., and *Stenotrophomonas* sp. YFMCD1 have been observed to degrade MC-LR. Meanwhile, *Sphingopyxis* sp. YF1 and *Bacillus* sp. AMRI-03 are reported to degrade MC-RR [11,12,13,14,15,16,17]. Additionally, the degradation of three MC variants (MC-RR, MC-YR, and MC-LR) by *Sphingopyxis* sp. MG-15 and *Novosphingobium* sp. MG-22 has been examined, with both strains being capable of degrading each variant. Notably, the degradation rate of MC-RR by these two strains was higher than that of MC-YR and MC-LR [18]. Furthermore, *Sphingomonas* sp. MD-1 has been shown to degrade three MCs, including MC-RR, MC-YR, and MC-LR, as indicated in the report on the degradation of a mixture of these three toxins [19]. However, it remains unclear how isolated bacteria can effectively degrade MCs in natural environments. The presence of these toxins in water bodies can negatively impact water quality, accumulate and magnify in food chains, and have adverse effects on plant, animal, and human health [20,21,22,23]. In Egypt, MCs have been detected in tilapia fish from tropical fishponds, with high concentrations found in the fish intestines, livers, and edible tissues. This poses significant health risks to humans and other organisms when these contaminated fish are consumed, particularly when intake exceeds tolerable daily levels [24]. Many patients died in Caruaru, Brazil, because they underwent hemodialysis with MC-contaminated water [25]. Therefore, effective degradation methods are required to remove the MCs from natural water.

Physical and chemical water treatments have been applied to natural water, but these methods are ineffective for removing MCs [26,27,28,29,30,31,32]. Water treatments such as ozone and chlorine treatments have been used to remove the MCs, but these methods have problems that harm byproducts, entail high running costs, and so on [33]. However, MCs can be degraded entirely using MC-degrading bacteria without harmful byproducts [33,34]. Among the MC degradation methods, biodegradation is helpful for MC control to demonstrate safety, highly efficient degradation, and cost-effectiveness [27] instead of physical and chemical water treatments. The first reported MC-degrading bacterium, *Sphingomonas* sp. ACM3962, was isolated from Murrumbidgee River, Australia [26]. After that, other MC-degrading bacteria have been isolated, most of which belong to the family Sphingomonadaceae [28,29,35,36,37,38,39,40,41]. Thus, the diversity of MC-degrading bacterial species is quite limited, and their degradation characteristics are still poorly understood.

The gene cluster responsible for the degradation of MCs, including *mlrA*, *mlrB*, *mlrC*, and *mlrD*, was identified by an assay using *Escherichia coli* [36,40,42]. The reports suggested that the *mlrA* gene encodes an enzyme for hydrolytic cleaving of the cyclic structure of MC-LR to linearize MC-LR as the first step. The enzymes encoded by *mlrB* and *mlrC* are responsible for further degrading linearized MC-LR into a tetrapeptide, as well as breaking down both linearized MC-LR and the tetrapeptide into Adda. Additionally, *mlrD* encodes a transporter protein that may facilitate active transport of MCs and their degradation products into cells. Maseda and Shimizu et al. revealed that *mlrA* is an essential gene responsible for MC degradation using *Sphingopyxis* sp. C-1 as the MC-degrading bacterium and analyzed a mutant with a disrupted *mlrA* gene [43]. Most MC-degrading bacteria contained the *mlrA* gene, as confirmed by PCR [11,12,14,41,44]. However, the *mlrA* gene was not detected in *Stenotrophomonas acidaminiphila* MC-LTH2, but this strain could effectively degrade MCs [45]. Therefore, we should check for the presence or absence of the *mlrA* gene if we isolate new MC-degrading bacteria.

Cyanobacterial blooms occur at different water temperatures from 15–30 °C worldwide [46]. In addition, the pH of water changes from neutral to alkaline due to photosynthesis by cyanobacteria. In Lake Kasumigaura, the pH value of water bodies reaches over 10.0 during the summer season when cyanobacterial blooms occur [19]. Therefore, alkaline-tolerant bacteria are required in eutrophicated lakes to degrade MCs. However, alkaline-tolerant bacteria are very limited among isolated MC-degrading bacteria. There are few studies that have explored the impacts of temperature, pH, and different MC variants on the biodegradation of MCs. The objectives of this study were to isolate new MC-degrading bacteria from Lake Kasumigaura and to investigate how temperature, pH, and MC variant affect MC biodegradation. Additionally, we examined the existence of the MC-degrading gene (*mlrA*) in three isolated strains.

## 2. Results

### 2.1. Isolation of MC-Degrading Bacteria from Lake Kasumigaura

**Figure 1:**
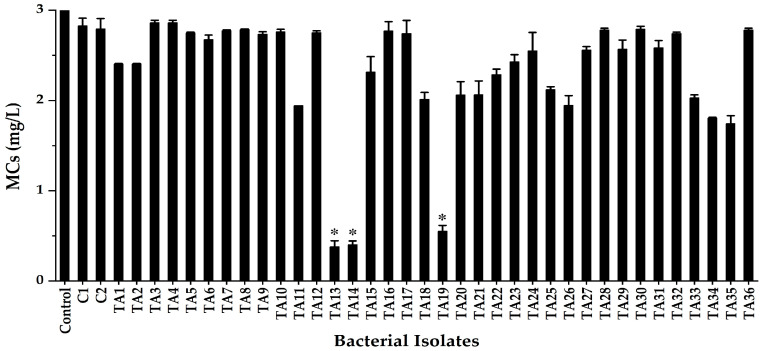
Screening of MC-degrading bacteria from water samples from Lake Kasumigaura. The error bars represent the standard deviation (SD) of the means of duplicates. The asterisk (*) indicates a significant difference between the control and the isolate, at *p* < 0.05.

A total of 38 colonies grown on mineral salt medium (MSM) containing MCs (1.0 mg/L) were isolated from Lake Kasumigaura (“35°52′–36°09′ N, 140°38′ E”) during HCBs. The MC-degradative ability of isolated colonies was investigated in peptone yeast extract (PY) broth with MCs (3.0 mg/L). Then, we selected three colonies, TA13, TA14, and TA19, which showed higher MC-degradative activity, for further analysis. The MC-degradative activities shown were 87.3% by TA13, 86.7% by TA14, and 81.6% by TA19 after 10 h incubation (Figure 1).

### 2.2. 16S rRNA Gene Sequencing Analysis

**Figure 2:**
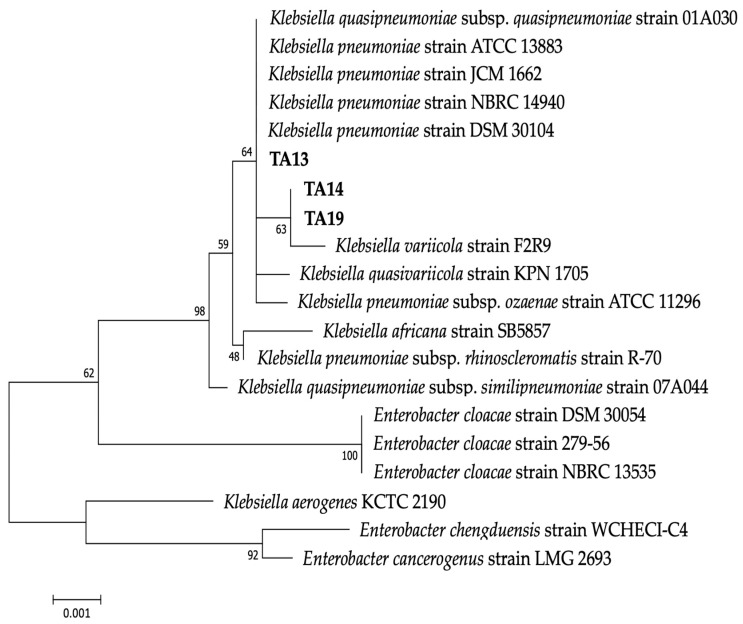
Phylogenetic tree of the isolated strains and closely related bacteria based on 16S rRNA gene sequencing. The tree was constructed using the neighbor-joining MEGA7 method. The numbers at branch nodes indicate the bootstrap values (1000 replications). The scale bar represents 0.001 substitutions per nucleotide position.

Single colonies of the three isolated strains are white in color and round-shaped. The colony diameter of strain TA13 was 3–4 mm, and that of strains TA14 and TA19 was 4–5 mm. Based on BLAST and phylogenetic analyses using 16S rRNA gene sequences, the three isolated strains were related to the genus *Klebsiella*, in which strain TA13 was most similar to *K. pneumoniae* strain DSM 30104 (99.7% similarity, accession number: NR_117683.1), and strains TA14 and TA19 most resembled *K. variicola* strain F2R9 (99.7% similarity, accession number: NR_025635.1) (Figure 2 and Table 1).

### 2.3. Effect of Temperature on the Biodegradation of MCs

**Figure 3:**
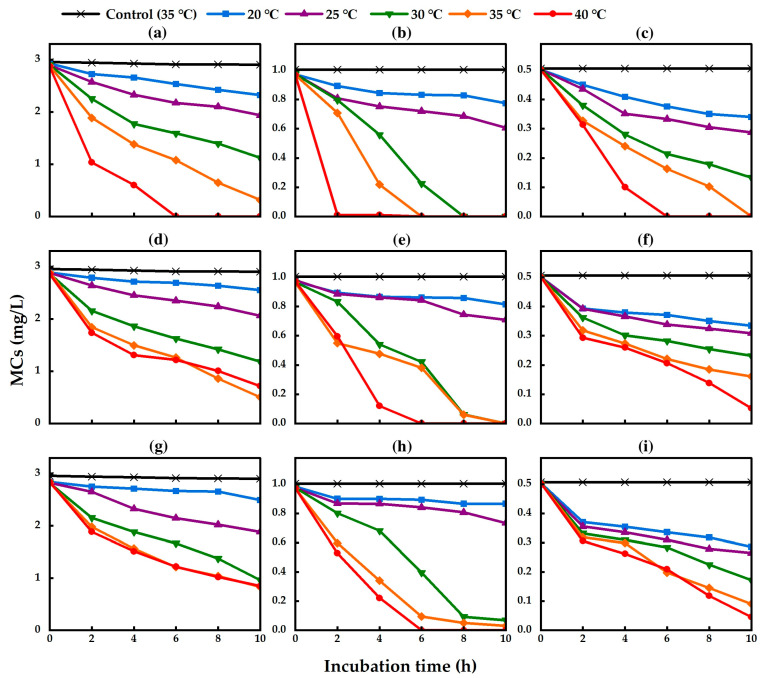
Effect of temperature on MC-degradative activity by isolated strains under different temperature conditions. (**a**) TA13 (MC-RR), (**b**) TA13 (MC-YR), (**c**) TA13 (MC-LR), (**d**) TA14 (MC-RR), (**e**) TA14 (MC-YR), (**f**) TA14 (MC-LR), (**g**) TA19 (MC-RR), (**h**) TA19 (MC-YR), and (**i**) TA19 (MC-LR).

MC biodegradation and degradative activity by isolated strains were examined in nutrient-free medium, which contained sterile Milli-Q water and MCs at different temperatures (20, 25, 30, 35, and 40 °C). The MC-degradative activity of the three isolated strains was measured by Ultra Performance Liquid Chromatography (UPLC). The standard curve of a MC quantified by UPLC is shown in Figure S1. The MC-degradative activity of TA13, TA14, and TA19 was increased by increasing the temperature from 20 °C to 40 °C (Figure 3). Strain TA13 completely degraded MC-RR at 40 °C within 6 h of incubation (Figure 3a and Figure S2). In addition, strain TA13 completely degraded MC-YR at 30 °C in 8 h, 35 °C in 6 h, and 40 °C in 2 h (Figure 3b and Figure S2). Furthermore, strain TA13 could completely degrade the MC-LR at 35 °C in 10 h and 40 °C in 6 h (Figure 3c and Figure S2). The strains TA14 and TA19 completely degraded MC-YR at 40 °C within 6 h of incubation (TA14; Figure 3e and Figure S3 and TA19; Figure 3h and Figure S4). No MCs were degraded in the control treatment without bacterial cells at 35 °C (Figure 3). The MC degradation rates by the isolated strains significantly increased at 35 °C and 40 °C. The highest degradation rates of MC-RR by strains TA13, TA14, and TA19 were 0.494 mg L^−1^ h^−1^, 0.239 mg L^−1^ h^−1^, and 0.227 mg L^−1^ h^−1^, those of MC-YR by strains TA13, TA14, and TA19 were 0.484 mg L^−1^ h^−1^, 0.243 mg L^−1^ h^−1^, and 0.142 mg L^−1^ h^−1^, and those of MC-LR by strains TA13, TA14, and TA19 were 0.126 mg L^−1^ h^−1^, 0.045 mg L^−1^ h^−1^, and 0.046 mg L^−1^ h^−1^, respectively, at 40 °C (Table 2).

### 2.4. Effect of pH on Bacterial Growth and Biodegradation of MCs

**Figure 4:**
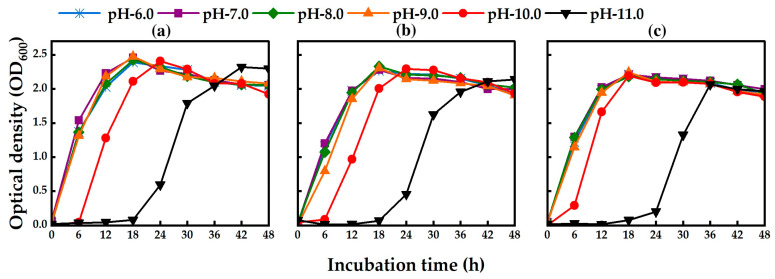
Effect of pH on the growth curves of the isolated strains. (**a**) TA13, (**b**) TA14, and (**c**) TA19.

**Figure 5:**
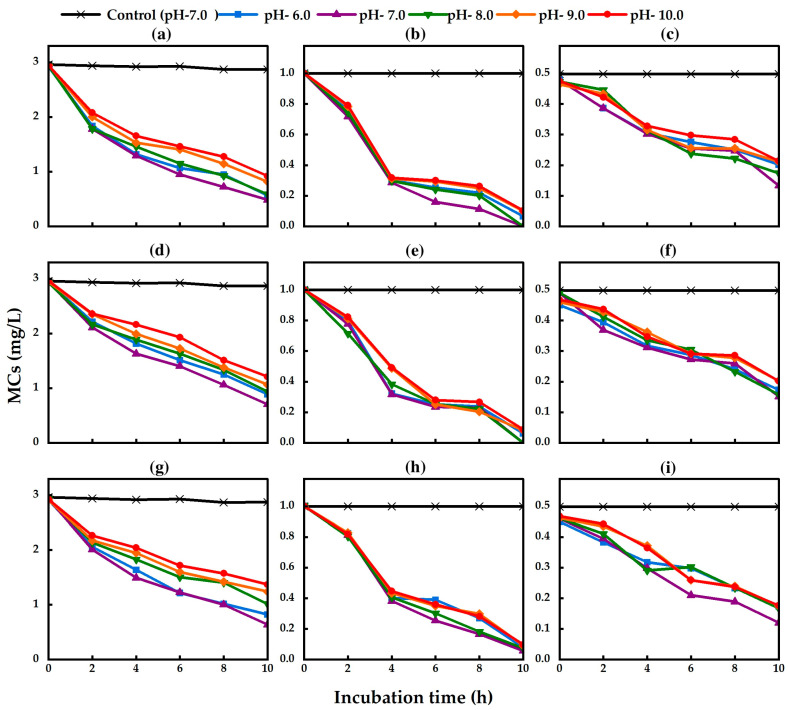
Effect of pH on MC-degradative activity by the isolated strains under different pH conditions. (**a**) TA13 (MC-RR), (**b**) TA13 (MC-YR), (**c**) TA13 (MC-LR), (**d**) TA14 (MC-RR), (**e**) TA14 (MC-YR), (**f**) TA14 (MC-LR), (**g**) TA19 (MC-RR), (**h**) TA19 (MC-YR), and (**i**) TA19 (MC-LR).

The isolated strains TA13, TA14, and TA19 could grow in PY broth medium under different pH conditions from 6.0 to 11.0 (Figure 4). There was no lag phase in the growth of the three isolated strains from pH 6.0 to 9.0. However, a lag phase was observed in the three isolated strains at pH 10.0. A longer lag phase was found at pH 11.0, but the bacteria could grow after 18 h of incubation (Figure 4a–c). These results indicated that the isolated strains could effectively grow under neutral and alkaline conditions. The isolated strains degraded MC-RR, MC-YR, and MC-LR under all pH conditions from 6.0 to 10.0 within 10 h of incubation (Figure 5). The pH values did not change in each treatment during the MC degradation experiment. There was no MC degradation in the control treatment group without bacterial cells (Figure 5). There were no significant differences in the degradation rates of MC by the isolated strains from pH 6.0 to 10.0. The highest degradation rates of MC-RR by strains TA13, TA14, and TA19 were 0.247 mg L^−1^ h^−1^, 0.224 mg L^−1^ h^−1^, and 0.229 mg L^−1^ h^−1^, those of MC-YR by strains TA13, TA14, and TA19 were 0.100 mg L^−1^ h^−1^, 0.100 mg L^−1^ h^−1^, and 0.095 mg L^−1^ h^−1^, and those of MC-LR by strains TA13, TA14, and TA19 were 0.035 mg L^−1^ h^−1^, 0.034 mg L^−1^ h^−1^, and 0.037 mg L^−1^ h^−1^, respectively, at pH 7.0 (Table 3). The isolated strains could also degrade the MCs under both neutral and alkaline conditions.

### 2.5. Detection of the mlrA Gene by PCR

**Figure 6:**
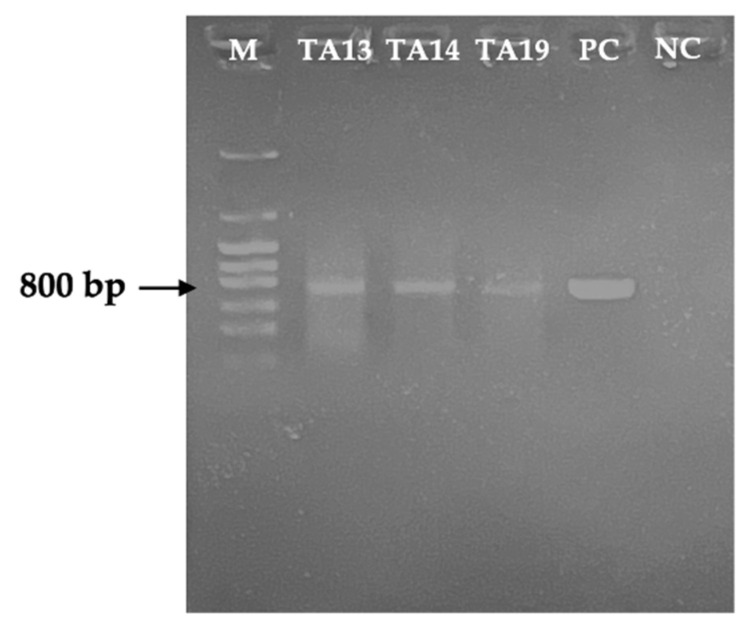
Detection of the *mlrA* gene in the isolated strains TA13, TA14, and TA19 by PCR, where M: pHY marker, PC: positive control (strain C-1), and NC: negative control.

Because isolated strains showed high MC-degradative activity, we tried detection of *mlrA* gene to check whether they possessed the *mlr* gene cluster by PCR analysis. As a result, the *mlrA* gene was detected in all three isolated strains (Figure 6).

### 2.6. Quantification of the mlrA Gene by Real-Time PCR (qPCR) Analysis

**Figure 7:**
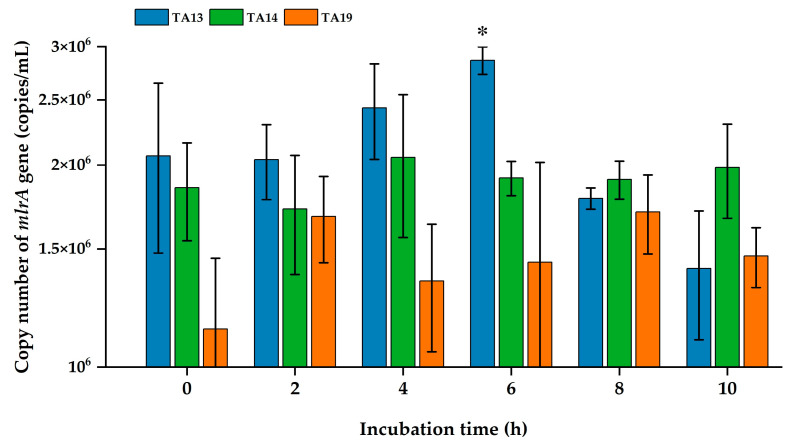
Copy numbers of the *mlrA* gene in isolated strains during MC biodegradation. The error bars represent the standard deviation (SD) of the means for triplicate samples. The asterisk (*) indicates significantly differences between incubation times, at *p* < 0.05.

*mlrA* gene expression in bacterial cells during MC biodegradation after 10 h of incubation was explored by real-time PCR analysis. The bacterial cell number of the isolated strains at the starting point (0 h incubation) was adjusted to 2.0 optical density units (OD_600_). The *mlrA* gene copy number of strain TA13 was significantly increased at 6 h and then declined at 8 h and 10 h of incubation (Figure 7). The copy number of *mlrA* gene in strains TA14 and TA19 was increased after adding the MCs. There were no statistically significant differences in the copy numbers of the *mlrA* gene during MC degradation.

## 3. Discussion

Because bacteria play a vital role in the biodegradation of MCs in natural lake water, bacterial MC biodegradation is a promising method to remove MCs from natural water without harming plant, animal, or human health and aquatic ecosystems. MC-degrading bacteria were isolated in previous studies [11,12,13,14,19,28,41,45,47,48]. However, few reports have analyzed MC-degradative activity at different temperatures and pH values with different MC analogs to examine MC-degradative activity during HCBs [19,28,45,48]. In this study, we isolated the three MC-degrading strains TA13, TA14, and TA19 from Lake Kasumigaura (Figure 1) and investigated the MC-degradative activity at different temperatures and pH values with different MC analogs (Figure 3 and Figure 5).

In this study, we identified three isolated strains belonging to the genus *Klebsiella* (Figure 2). Only one report has related *Klebsiella* sp. YFMCD1 to MC biodegradation, but YFMCD1 was not a single isolated strain (it was a combination of *Klebsiella* sp. and *Stenotrophomonas* sp.) [15]. So, this is the first report of an isolated *Klebsiella* sp. capable of degrading MCs. In this study, we successfully isolated a new *Klebsiella* sp. as a single strain that can degrade MCs. Shao et al., Zhang et al., and Qiu et al. have shown that *Klebsiella* spp. are highly efficient at biodegradation of tetracycline, tylosin, and terephthalate (TPA) compounds [49,50,51].

Temperature and pH are crucial factors in the occurrence of cyanobacterial blooms and significantly impact the biodegradation of MCs. For example, MC-LR and MC-RR degradation rates were strongly influenced by incubation temperature, and the degradation rate of MC by *Sphingomonas* sp. Y2 increases as the temperature rises from 5 °C to 30 °C [47]. Many studies indicate that the optimum temperature for MC degradation is 30 °C, and the highest degradation rates are observed at 30 °C under neutral conditions [13,14,19,47,48]. However, the bacterial community YFMCD1 (*Klebsiella* sp. and *Stenotrophomonas* sp.) degraded MC-LR at 30 °C and 40 °C [15]. In this study, the isolated strains effectively degraded MCs at temperatures of 30, 35, and 40 °C within 10 h of incubation (Figure 3). Strain TA13 completely degraded MCs at 40 °C (Figure 3a–c). Moreover, strain TA13 had the highest MC-degradative activity among the three strains. All of the strains showed a significant increase in MC degradation rates at 35 °C and 40 °C, and the highest degradation rates were observed at 40 °C (Table 2). This result indicated that bacterial metabolism was very active at high temperature and produced the enzymes for MC degradation [11]. Enhancing the MC degradation rate at high temperatures could potentially control increasing the production of MCs during cyanobacterial blooms, especially in warm weather conditions [52].

For MC-RR degradation, the three isolated strains had higher degradation rates (TA13; 0.494 mg L^−1^ h^−1^, TA14; 0.239 mg L^−1^ h^−1^, and TA19; 0.227 mg L^−1^ h^−1^) at 40 °C compared to the degradation rates by *Bacillus* sp. AMRI-03 (0.060 mg L^−1^ h^−1^) at 28 °C, *Bacillus* sp. SSZ01 (0.10 mg L^−1^ h^−1^), and *Bordetella* sp. MC-LTH1 (0.17 mg L^−1^ h^−1^) at 30 °C [17,41,48], but lower than *Sphingomonas* sp. Y2 (0.542 mg L^−1^ h^−1^) at 30 °C [47] and *Sphingopyxis* sp. YF1 (0.770 mg L^−1^ h^−1^) at 30 °C [16]. For MC-YR degradation, strains TA13, TA14, and TA19 had higher degradation rates (0.484 mg L^−1^ h^−1^, 0.243 mg L^−1^ h^−1^, and 0.142 mg L^−1^ h^−1^, respectively) compared to the degradation rates by *Sphingopyxis* sp. MG-15 (0.026 mg L^−1^ h^−1^) and *Novosphingobium* sp. MG-22 (0.006 mg L^−1^ h^−1^) [18]. For MC-LR degradation, the isolated strains had higher degradation rates (TA13; 0.126 mg L^−1^ h^−1^, TA14; 0.045 mg L^−1^ h^−1^, and TA19; 0.046 mg L^−1^ h^−1^, respectively) than *Sphingopyxis* sp. m6 (0.003 mg L^−1^ h^−1^ at 30 °C and 0.002 mg L^−1^ h^−1^ at 37 °C) [14], but lower than *Sphingomonas* sp. Y2 (0.225 mg L^−1^ h^−1^) at 30 °C [47]. The optimum temperature of MC-degrading bacteria in previous studies was observed to be 30 °C [11,14,28,45,47,48]. In this study, the three isolated strains showed high growth rates at 35 °C and 40 °C, and the highest growth was observed at 40 °C (Figure S5). The highest MC degradation by the isolated strains was also observed at 40 °C. These findings showed that the three isolated strains could degrade the MCs at higher temperatures compared to previously isolated MC-degrading bacteria.

In aquatic environments, pH changes in natural water from neutral to alkaline conditions occur during HCBs. Therefore, checking the effect of different pH conditions is very important for biodegradation of MCs by isolated strains [34]. In this study, the three isolated strains could grow not only under neutral conditions but also under alkaline conditions (Figure 4). In a previous study, *Sphingopyxis* sp. strain C-1 efficiently degraded MCs from pH 6.0 to 9.5 [34], and *Klebsiella* sp. and *Stenotrophomonas* sp. strain YFMCD1 degraded MC-LR from pH 5.0 to 11.0 [15]. The highest MC-LR degradation activity of *Sphingomonas* sp. strain MD-1 was observed at pH 7.2, but degradation activity was decreased under alkaline conditions (pH 9.5) [19]. In this study, the isolated strains could grow well and effectively degrade MCs at pH values of 6.0 to 10.0, and the optimum pH for MC degradation was pH 7.0. We concluded that the three isolated strains are alkaline-tolerant bacteria capable of MC degradation. Shao et al. reported that *Klebsiella* sp. SQY5 degraded tetracycline at temperatures of 30 °C and 35 °C and pH values of 5.0 to 10.0. Strain SQY5 could also degrade tetracycline at 40 °C and pH 10.0 [49]. The highest degradation efficiency of tylosin by *Klebsiella pneumoniae* TN-1 was at 37 °C and pH 7.0 after 7 days’ incubation [50]. Moreover, the highest terephthalate (TPA) degradation rate of *Klebsiella variico* SY1 was observed at 30 °C within 24 h incubation, and strain SY1 could also degrade TPA at 37 °C [51].

For MC-RR degradation, that of strain TA13 (0.247 mg L^−1^ h^−1^) was higher than that of *Stenotrophomonas acidaminiphila* MC-LTH2 (0.233 mg L^−1^ h^−1^), but those of strains TA14 and TA19 (0.224 mg L^−1^ h^−1^ and 0.229 mg L^−1^ h^−1^, respectively) were slightly lower than that of MC-LTH2 at pH 7.0 after 7 days’ incubation [45]. For MC-YR degradation, the isolated strains had a higher degradation rate (TA13; 0.100 mg L^−1^ h^−1^, TA14; 0.100 mg L^−1^ h^−1^, and TA19; 0.095 mg L^−1^ h^−1^) at pH 7.0 compared to the degradation rates by *Sphingopyxis* sp. MG-15 (0.004 mg L^−1^ h^−1^) and *Novosphingobium* sp. MG-22 (0.003 mg L^−1^ h^−1^) at pH 7.6 [18]. For MC-LR degradation, strains TA13 (0.035 mg L^−1^ h^−1^), TA14 (0.034 mg L^−1^ h^−1^), and TA19 (0.037 mg L^−1^ h^−1^) exhibited higher degradation rates than *Sphingopyxis* sp. m6 (0.003 mg L^−1^ h^−1^) at pH 7.0 [14]. However, these strains exhibited lower rates than *Stenotrophomonas acidaminiphila* MC-LTH2 (0.125 mg L^−1^ h^−1^) [45] and YFMCD1 (*Klebsiella* sp. and *Stenotrophomonas* sp.) (0.25 mg L^−1^ h^−1^) at the same pH [15]. Some isolated bacteria from previous reports, such as *Sphingpoyxis* sp. [13,14,16], *Sphingomonas* sp. [11,19], *Bacillus* sp. [17], *Stenotrophomonas* sp. [45], and *Bordetella* sp. [48], degraded MCs under neutral pH conditions (pH 7.0). In this study, *Klebsiella* sp. strains TA13, TA14, and TA19 degraded MCs under both neutral and alkaline conditions.

This study also reveals that the three strains had the MC-degrading gene (*mlrA*), which encodes a hydrolytic enzyme responsible for cleaving the cyclic structure of MC. Detection of the *mlrA* gene in these three strains suggests that degradation of MCs by *Klebsiella* sp. follows the MC-LR degradation pathway identified in previous studies [36,42]. The presence of the *mlrA* gene in genus *Klebsiella* supports the hypothesis that this gene is uniquely responsible for degrading MCs, unlike in the genera *Sphingomonas* and *Sphingobium* [11,14,34,44]. Additionally, homologous sequences of the *mlrA* gene were detected in *Bacillus* sp. AMRI-03 and SSZ01 and *Bordetella* sp. MC-LTH1, which are known to degrade MC-RR and MC-LR [17,41,48]. This is the first report identifying the *mlrA* gene in genus *Klebsiella*.

The *mlrA* gene copy number of strain TA13 was increased compared to strains TA14 and TA19 from 2 h to 6 h of incubation (Figure 7). In the MC degradation test, the MC-degradative activity of strain TA13 was higher than that of strains TA14 and TA19 at 40 °C (Figure 3). Shimizu et al. demonstrated that expression of the *mlrA* gene by *Sphingopyxis* sp. C-1 significantly increased within 30 min of adding MC [53]. Furthermore, the MC-degrading bacterium *Sphingopyxis* sp. m6 showed an increase in *mlrA* gene expression at 1 h, followed by a decline from 2 to 6 h [14]. In our three isolated strains, particularly strain TA13, the *mlrA* gene copy number significantly increased at 6 h, then decreased by 8 h and 10 h. A similar pattern of *mlrA* gene expression in strain TA13 was found in a previous study related to MC degradation [14,53]. This result illustrated that MCs enhance the activity of MC-degrading enzymes and stimulate *mlrA* gene expression during MC degradation [54].

## 4. Conclusions

In this study, we successfully isolated three strains of *Klebsiella* spp., TA13, TA14, and TA19, from Lake Kasumigaura that exhibit significant activity in degrading MCs. These isolated strains can grow effectively and degrade MCs efficiently at mesophilic temperatures and under alkaline conditions. The highest rates of MC degradation were observed at a temperature of 40 °C and a pH of 7.0. Furthermore, all three strains possess the *mlrA* gene, which is crucial for the degradation of MCs. This finding indicates that these strains follow a typical pathway for MC degradation. Our results suggest that TA13, TA14, and TA19 can degrade MCs at higher temperatures and a wider range of pH levels than previously isolated MC-degrading bacteria. Therefore, these three strains of *Klebsiella* spp. have the potential to be used for bioremediation of MCs in both temperate and tropical aquatic environments. They can rapidly and effectively degrade various MC variants (MC-RR, MC-YR, MC-LR) at high water temperatures and under elevated pH conditions, thus enhancing their applicability in natural lake waters as global temperatures rise.

## 5. Materials and Methods

### 5.1. Water Sample Collection

In Lake Kasumigaura, water blooms occur every year from July to October [55]. In 1982, *Microcystis aeruginosa* NIES-102 was isolated from Lake Kasumigaura, and this strain mainly produced MC-RR, MC-YR, and MC-LR [56]. In August 2021, surface water samples were collected in 0.5 L sterile glass bottles from three sampling points in Lake Kasumigaura (Tsuchiura-iri, Lake Center, and Takahama-iri). These collected water samples were kept on ice (less than 12 h), and then the MC-degrading bacteria were isolated.

### 5.2. Isolation of MC-Degrading Bacteria from Water Samples

The collected water samples were filtered through a membrane filter (5.0 µm pore size). The filtered samples (100 mL) were amended with NH_4_NO_3_ and KH_2_PO_4_ (0.05 mM as a final concentration) and incubated at 28 °C with shaking at 150 rpm for 48 h in the dark. The incubated samples (100 µL) were spread on MSM medium containing (in g/L) KH_2_PO_4_ 1.36, (NH_4_)_2_SO_4_ 0.5, MgSO_4_7H_2_O 0.2, CaCl_2_2H_2_O 0.01, FeSO_4_7H_2_O 0.005, MnSO_4_7H_2_O 0.003, Na_2_MoO_4_2H_2_O 0.003, Na_2_HPO_4_ 0.002, and agar 15.0, and then the inoculated plates were incubated at 28 °C for 48 h. Single colonies from these plates were transferred to new MSM grid-numbered plates containing MCs (1.0 mg/L) (see Section 5.4). This experiment was carried out in triplicate. Finally, a total of 38 colonies were collected for screening for MC-degrading bacteria.

For investigation of their MC-degradative activity, these collected colonies were pre-cultured into PY broth medium containing (in g/L) peptone 10.0 and yeast extract 5.0, pH 7.0) and then incubated at 30 °C with shaking at 200 rpm for 18 h. The bacterial cells were harvested by centrifugation at 6500× *g* for 5 min and washed two times with 0.1 M potassium phosphate buffer. After that, MCs (3.0 mg/L) were mixed into sterile Milli-Q water and inoculated into the suspended bacterial cells into each test tube (total 5.0 mL). A sample without bacterial cells was used as a control. The inoculated test tubes were cultured at 30 °C with shaking at 200 rpm. Samples (300 µL) were withdrawn at 2 h intervals, and MC concentrations were measured in the sub-samples by UPLC analysis [28]. This experiment was carried out in duplicate. The isolated strains were preserved in PY broth with 40% (*v*/*v*) glycerol stock and stored at −80 °C.

### 5.3. Identification of Isolated MC-Degrading Bacteria

The isolated bacteria were identified using 16S rRNA gene sequences and phylogenetic characteristics. The colony color, size, and shape of the isolated strains were observed on PY solid medium after 24 h of incubation. The 16S rRNA gene sequences were determined using the primers 27F: 5′-AGAGTTTGATCMTGGCTCAG-3′ and 1492R: 5′-CGGTTACCTTGTTACGACTT-3′ by PCR. The PCR-amplified products were sequenced using ABI 3730xl DNA Analyzer (Applied Biosystems, Applied Biosystems, USA) (Seibutsu Giken Co., Ltd., Japan). To determine and compare the homology of the 16S rRNA sequences, we utilized the Basic Local Alignment Search Tool (BLAST) in the National Center for Biotechnology Information (NCBI) database (https://blast.ncbi.nlm.nih.gov/Blast.cgi, accessed on 17 October 2023). Multiple sequences were aligned using ClustalW, and a phylogenetic tree was constructed using the neighbor-joining method by MEGA7 software [57]. The sequences were deposited in the DNA Data Bank of Japan (DDBJ) with accession numbers LC791357 to LC791359.

### 5.4. Purification and Analysis of MCs

*M. aeruginosa* NIES-102 was obtained from the National Institute of Environmental Studies (NIES) culture collection. This strain was cultured using M-11 liquid medium containing (in g/L) NaNO_3_ 0.1, K_2_HPO_4_ 0.01, MgSO_4_7H_2_O 0.075, CaCl_2_2H_2_O 0.04, Na_2_CO_3_ 0.03, FeSO_4_7H_2_O 0.001, and Na_2_EDTA2H_2_O 0.001 at pH 8.0 [58]. MCs were extracted from the *M. aeruginosa* culture and purified according to the previously reported method [59]. *Microcystis* cells were suspended into 5% acetic acid and shaken at 150 rpm for 20 min. These extracted mixtures were centrifuged at 12,000× *g* for 20 min, then the supernatant was used to purify the MCs using a Presep^®^-C C18 (ODS) Cartridge (Fujifilm Wako Pure Chemical Corporation, Osaka, Japan). A C18 (ODS) Cartridge was pre-conditioned with 100% methanol and Milli-Q water. The extracted samples were passed through the C18 (ODS) Cartridge and then rinsed with 20% methanol and Milli-Q water to remove small-sized solids. After that, the MCs adsorbed onto the cartridge were eluted with 100% methanol and evaporated using nitrogen gas at 40 °C in a water bath. After evaporation, the dry solid MCs were re-dissolved in 100% methanol and diluted with sterile Milli-Q water. These samples were filtered through a 0.2 μm Millex^®^ syringe filter (Millex^®^—LG, Merck Millipore Ltd., Japan), and the filtrates were transferred into sample vials to determine the MC concentration by UPLC. The extracted MCs were used for investigation of MC degradation ability by isolated bacteria. Standard MCs (MC-RR, MC-YR, and MC-LR) were purchased from Fujifilm Wako Pure Chemical Industries Ltd. (Osaka, Japan) to determine the concentrations of the extracted MCs. The extracted samples and standard MCs were analyzed by UPLC (ACQUITY™ UPLC^®^, Waters, Milford, MA, USA) using an ultraviolet (UV) detector set at 238 nm for MC detection equipped with a reverse-phase C18 column (ACQUITY UPLC^®^ BEH C18 (1.7 µm, 2.1 mm × 100 mm, Waters, USA). Two mobile phases were applied, including mobile phase A (100% methanol) and mobile phase B (phosphorous buffer pH 2.5 (*v*/*v*)). The applied analytical procedure was gradient run as follows: 0 min and 55% A, 7 min and 56% A, 7 min 1 s and 80% A, 9 min and 80% A, 9 min 1 s and 55% A, 12 min and 55% A. The temperatures of the samples and column were 10 °C and 40 °C, respectively. The flow rate was 0.200 mL/min. The injection volume of the sample was 10.0 µL.

### 5.5. Effect of Temperature on MC Biodegradation

The bacterial strains were cultured in PY broth medium and incubated at 30 °C with shaking at 200 rpm for 18 h. The bacterial cells were harvested by centrifugation at 6500× *g* for 5 min at 4 °C and were washed with 0.1 M phosphate buffer solution. This washing process was repeated twice, and the bacterial cells were suspended in the buffer solution. After that, MCs (3.0 mg/L) were added to sterilized Milli-Q water (pH 7.0) and inoculated into the suspended bacterial culture. The culture volume was set at 5.0 mL. These inoculated test tubes were incubated at various temperatures (20, 25, 30, 35, and 40 °C) with shaking at 200 rpm. A bacteria-free sample (35 °C) was used as the control. Samples (300 µL) were taken at intervals (0 h, 2 h, 4 h, 6 h, 8 h, and 10 h), and the remaining MC concentration was analyzed by UPLC to determine the MC-degradative activity of the isolated bacteria. This experiment was carried out in triplicate.

### 5.6. Effect of pH on Growth of the Isolated Bacteria

Growth of the isolated strains TA13, TA14, and TA19 was examined under different pH conditions. The bacterial cells were pre-cultured in PY broth medium (pH 7.0) and then incubated at 30 °C with shaking at 200 rpm. After 3 days of incubation, 25 µL of incubated culture (200 times dilution, OD value = 0.01) was inoculated into PY broth (5.0 mL) with pH values ranging from 6.0 to 11.0. The pH levels were adjusted using 1.0 M hydrochloric acid (HCl) and 1.0 M sodium hydroxide (NaOH). The cultured test tubes were incubated at 30 °C with shaking at 200 rpm. The optical density (OD) was measured at 600 nm every 6 h using a spectrophotometer (Shimadzu UV Mini-1240 UV-VIS Spectrophotometer, Kyoto, Japan). This experiment was carried out in triplicate.

### 5.7. Effect of pH on MC Biodegradation

The MC-degradative activity of the isolated bacteria was examined at different pH values (6.0, 7.0, 8.0, 9.0, and 10.0). The samples were prepared in the same way as described in Section 5.5. The inoculated test tubes were cultured at 35 °C with shaking at 200 rpm. A bacteria-free sample (pH 7.0) was used as a control. Samples (300 µL) were taken at intervals (0 h, 2 h, 4 h, 6 h, 8 h, and 10 h), and the residual MC concentration was analyzed by UPLC to determine the MC-degradative activity of the isolated bacteria. After 10 h of incubation, the pH value in each test tube was measured to check the stability. This experiment was carried out in triplicate.

### 5.8. Detection of the mlrA Gene in MC-Degrading Bacteria

The genomic DNA of the isolated bacteria was extracted from fresh culture plates using an ISOIL for Beads Beating kit (Nippon Gene, Osaka, Japan) according to the manufacturer’s protocol. Two specific primers, MF: 5′-CCCGATGTTCAAGATACT-3′ and MR: 5′-CTCCTTCCACAAATCAGGAC-3′ [44], were used to detect the presence of the *mlrA* gene in the isolated strains by PCR using Takara Ex Taq (Takara Bio Inc., Kyoto, Japan), with a PCR product of approximately 800 bp. The PCR reaction mixture was prepared in a total volume of 20 μL, containing 2 µL of 20 mM of 10 × Ex Taq Buffer, 1.6 µL of 2.5 mM of dNTPs, 0.1 µL of 0.5 U of Ex Taq polymerase, 0.5 µL of 10 µM of MF primer, 0.5 µL of 15 µM of MR primer, 1 µL of extracted DNA, and 14.3 µL of sterile Milli-Q water. The PCR assay was performed under the following conditions: initial denaturation at 94 °C for 5 min, followed by 40 cycles of denaturation for 30 s at 94 °C, annealing for 30 s at 50 °C, and extension for 2 min at 72 °C, and final elongation for 7 min at 72 °C. In this study, *Sphingopyxis* sp. C-1 was used as a positive control to detect the *mlrA* gene. The PCR products were analyzed by 1% agarose gel electrophoresis at 50 V for 40 min and visualized using an UV transilluminator.

### 5.9. Quantification of the mlrA Gene During MC Degradation

Preparation of bacterial cells with MCs was conducted following the same method as described in Section 5.5 regarding the effect of temperature on MC degradation. The inoculated test tubes were cultured at 40 °C. Samples of the incubated bacterial cultures (500 μL) were taken at intervals during MC biodegradation. These incubated cultures were centrifuged at 12,000× *g* for 10 min at 4 °C. After centrifugation, bacterial cell pellets were used to extract DNA for qPCR analysis. The DNA was extracted using an ISOIL for Beads Beating kit (Nippon Gene, Osaka, Japan) following the manufacturer’s protocol. The total DNA was suspended in 50 μL of TE buffer and stored at –30 °C. A qPCR assay was performed using an Applied Biosystems^®^ 7500 Real-Time PCR system. For this analysis, two specific primers, qmlrAf: 5′-AGCCCKGGCCCRCTGC-3′ and qmlrAr: 5′-ATGCCARG CCCACCACAT-3′ [60], were used to quantify the copy number of the *mlrA* gene. The qPCR reaction mixture was prepared in a total volume of 20 μL, containing 10 μL of SYBR^®^ qPCR mix (THUNDERBIRD™), 0.5 μL of each 10 μM primer, 0.4 μL of 50x ROX reference dye, 1 μL of extracted DNA, and 7.6 μL of sterilized Milli-Q water. The reaction conditions were initial denaturation at 95 °C for 4 min and 40 cycles of denaturation at 95 °C for 20 s, annealing at 55 °C for 40 s, and extension at 72 °C for 1 min with fluorescence acquisition, followed by melting curve analysis. For calibration, a DNA standard curve for the *mlrA* gene was prepared by serial dilution of a purified *mlrA* gene fragment from strain C-1. The copy number of the *mlrA* gene for the standard curve was calculated using the following formula [61]:

Number of copies (copies/μL) = Concentration of DNA (ng/μL) × 10^−9^ × 6.022 × 10^23^/Length of DNA (bp) × 660.

### 5.10. Statistical Analysis

The data obtained in this experiment were analyzed by one-way analysis of variance (ANOVA) using IBM SPSS software (Version 28.0). Mean values were compared to the least significant difference (LSD) value according to Scheffe’s test (*p* < 0.05).

## Figures and Tables

**Table 1:** BLAST analysis of the 16S rRNA gene sequences of isolated strains.

Strain	Gene Bank Accession No.	Closely Related sp.	Strain No.	Sequence (bp)	Similarity (%)
TA13	LC791357	*Klebsiella pneumoniae* (NR_117683.1)	DSM 30104	1454/1459	99.7
TA14	LC791358	*Klebsiella variicola* (NR_025635.1)	F2R9	1408/1412	99.7
TA19	LC791359	*Klebsiella variicola* (NR_025635.1)	F2R9	1408/1412	99.7

**Table 2:** Effect of temperature on the MC degradation rate by isolated strains.

Strain	Degradation Rate (mg L^−1^ h^−1^)														
	20 °C			25 °C			30 °C			35 °C			40 °C		
	MC-RR	MC-YR	MC-LR	MC-RR	MC-YR	MC-LR	MC-RR	MC-YR	MC-LR	MC-RR	MC-YR	MC-LR	MC-RR	MC-YR	MC-LR
TA13	0.064 a	0.020 a	0.016 a	0.110 ab	0.037 a	0.021 ab	0.194 bc	0.122 b	0.037 bc	0.293 c	0.161 b	0.050 c	0.494 d	0.484 c	0.126 d
TA14	0.047 a	0.016 a	0.019 a	0.099 ab	0.027 a	0.019 a	0.175 bc	0.109 b	0.027 ab	0.229 c	0.109 b	0.034 b	0.239 c	0.243 c	0.045 c
TA19	0.046 a	0.012 a	0.022 a	0.104 ab	0.024 a	0.024 a	0.196 bc	0.103 ab	0.033 ab	0.207 bc	0.107 ab	0.041 b	0.227 c	0.142 b	0.046 b

The different letters shown after the data represent significant differences between the temperatures for the same target MC. Numbers followed by the same letter do not differ significantly in LSD value at *p* < 0.05.

**Table 3:** Effect of pH on the MC degradation rate by isolated strains.

Strain	Degradation Rate (mg L^−1^ h^−1^)														
	pH 6.0			pH 7.0			pH 8.0			pH 9.0			pH 10.0		
	MC-RR	MC-YR	MC-LR	MC-RR	MC-YR	MC-LR	MC-RR	MC-YR	MC-LR	MC-RR	MC-YR	MC-LR	MC-RR	MC-YR	MC-LR
TA13	0.236 ab	0.094 a	0.028 a	0.247 b	0.100 a	0.035 a	0.235 ab	0.100 a	0.030 a	0.213 ab	0.090 a	0.026 a	0.203 a	0.090 a	0.026 a
TA14	0.205 ab	0.094 a	0.032 a	0.224 b	0.100 a	0.034 a	0.200 ab	0.100 a	0.033 a	0.188 ab	0.092 a	0.029 a	0.176 a	0.092 a	0.029 a
TA19	0.209 a	0.092 a	0.032 a	0.229 a	0.095 a	0.037 a	0.192 a	0.093 a	0.032 a	0.167 a	0.091 a	0.032 a	0.156 a	0.091 a	0.031 a

The different letters shown after the data represent significant differences between the pH for the same target MC. Numbers followed by the same letter do not differ significantly in LSD value at *p* < 0.05.

## Data Availability

The original data presented in the study are openly available in the DNA Data Bank of Japan (DDBJ) [accession numbers: LC791357 to LC791359].

## Supplementary Materials

The following supporting information is available at https://www.mdpi.com/article/10.3390/toxins17070346/s1, Figure S1: Calibration curve of a standard MC quantified by UPLC; Figure S2: Chromatograms of UPLC analysis of MC degradation at different temperatures and pH values for strain TA13 at 40 °C and pH 7.0 (a) 0 h, (b) 2 h, (c) 4 h, (d) 6 h, (e) 8 h, (f) 10 h; Figure S3: Chromatograms of UPLC analysis of MC degradation at different temperatures and pH values for strain TA14 at 40 °C and pH 7.0 (a) 0 h, (b) 2 h, (c) 4 h, (d) 6 h, (e) 8 h, (f) 10 h; Figure S4: Chromatograms of UPLC analysis of MC degradation at different temperatures and pH values for strain TA19 at 40 °C and pH 7.0 (a) 0 h, (b) 2 h, (c) 4 h, (d) 6 h, (e) 8 h, (f) 10 h; Figure S5: Effect of temperature on growth of the isolated strains (a) TA13, (b) TA14, and (c) TA19.
