# Supplementary material for: Biodegradation of Microcystins by Aquatic Bacteria Klebsiella spp. Isolated from Lake Kasumigaura
Source: Toxins (Basel). 2025 Jul 10;17(7):346. doi: 10.3390/toxins17070346 (PMC12298343; doi:10.3390/toxins17070346)
Supplement: Supplementary file 1 [file toxins-17-00346-s001.zip › toxins-3679428-supplementary.pdf]

toxins

MDPI

Supplementary Materials: Biodegradation of Microcystins by Aquatic Bacteria *Klebsiella* sp. Isolated from Lake Kasumigaura

Thida Lin, Kazuya Shimizu, Tianxiao Liu, Qintong Li, and Motoo Utsumi

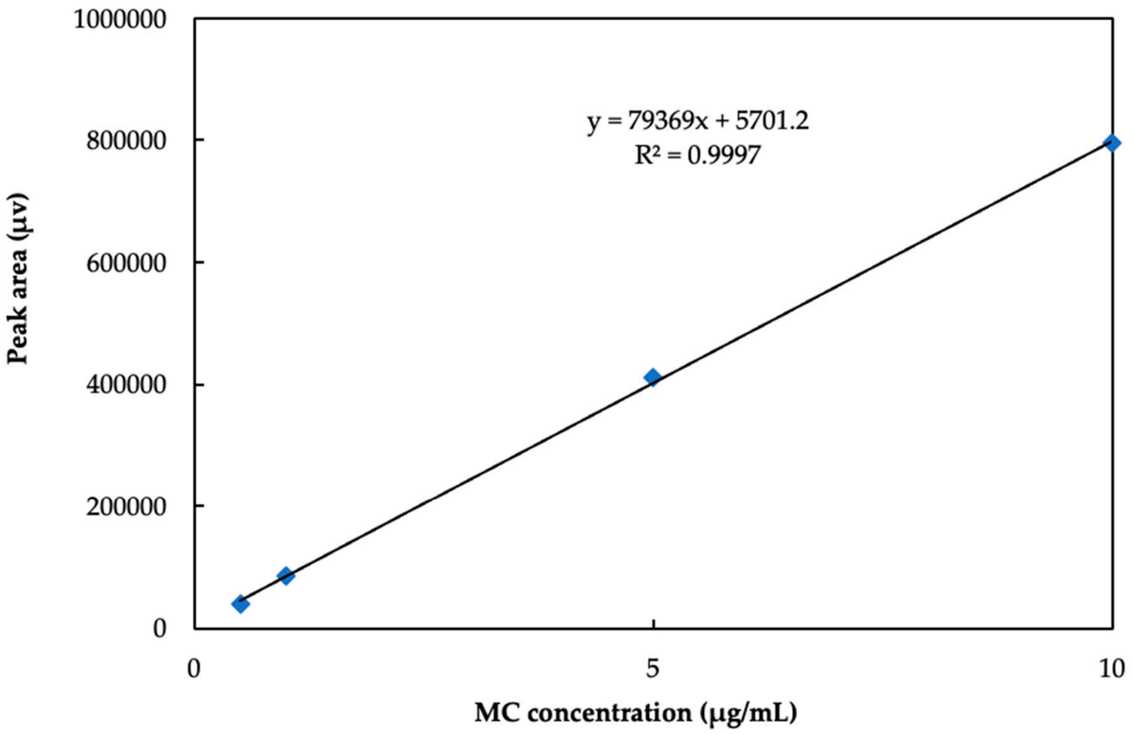

Figure S1. Calibration curve of a standard MC quantified by UPLC.

 https://doi.org/10.3390/toxins17070346

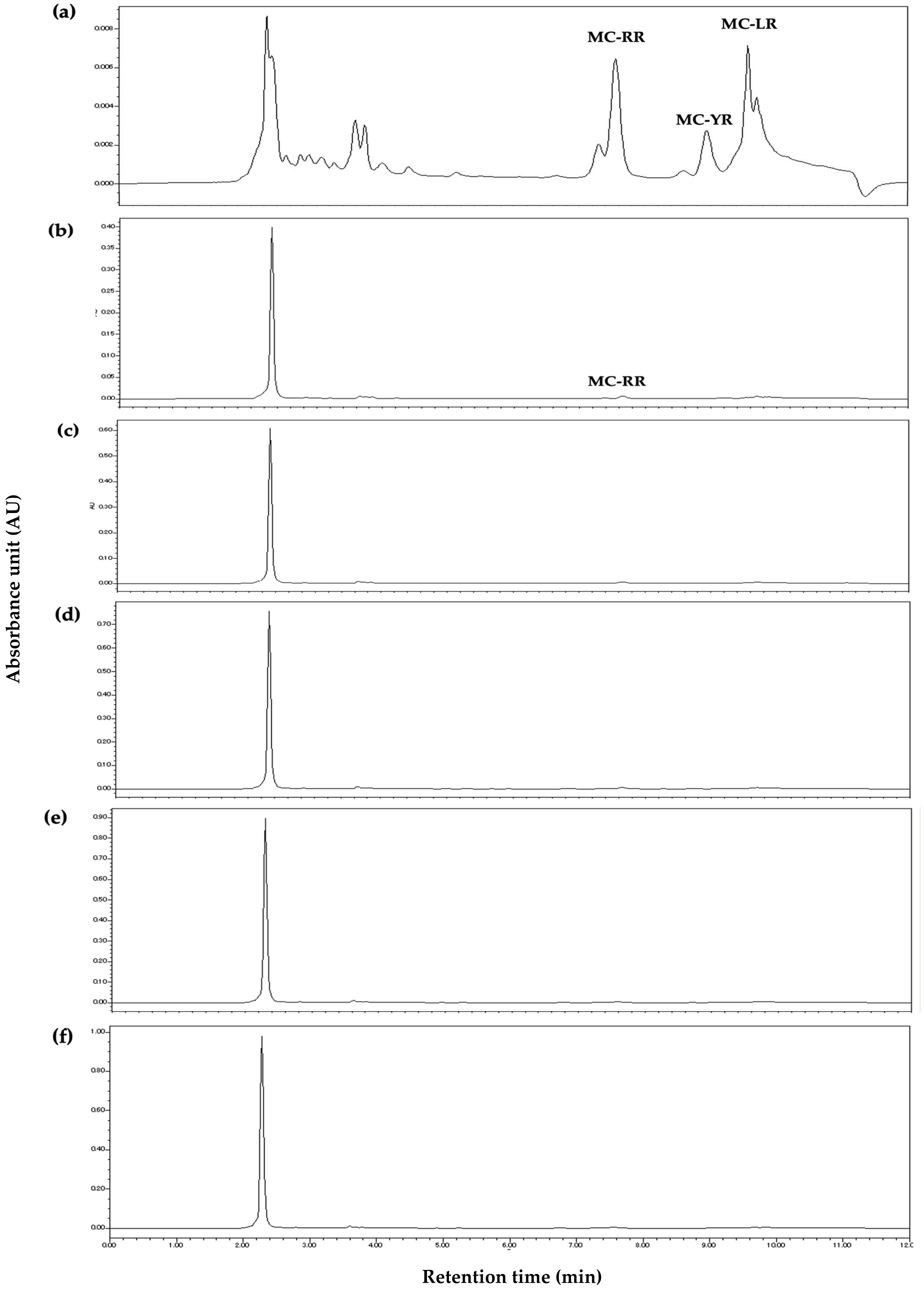

Figure S2. Chromatograms of UPLC analysis of for MC degradation at different temperatures and pH values for strain TA13 at 40 °C and pH 7.0 (a) 0 h, (b) 2 h, (c) 4 h, (d) 6 h, (e) 8 h, (f) 10 h.

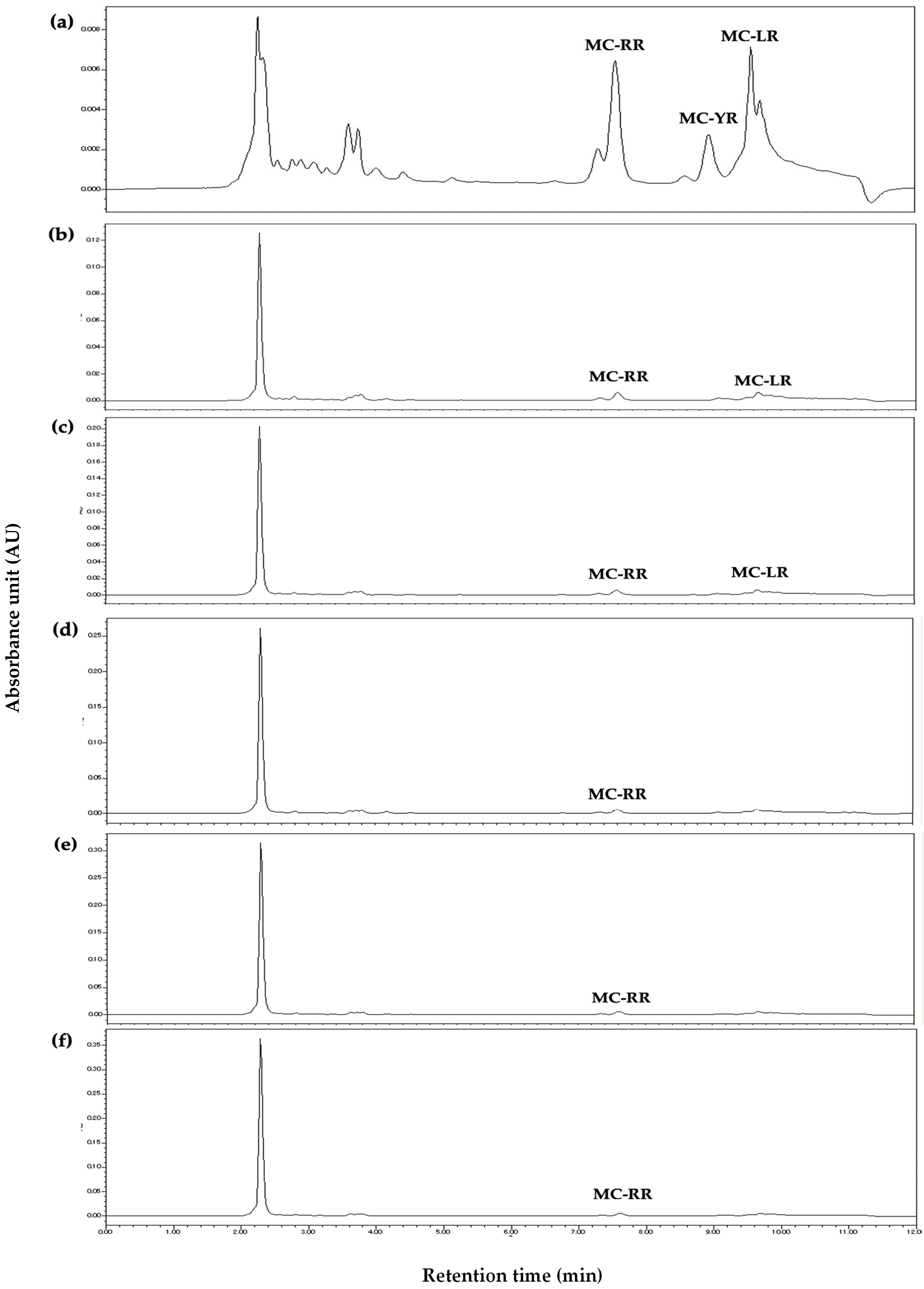

Figure S3. Chromatograms of UPLC analysis of for MC degradation at different temperatures and pH values for strain TA14 at 40 °C and pH 7.0 (a) 0 h, (b) 2 h, (c) 4 h, (d) 6 h, (e) 8 h, (f) 10 h.

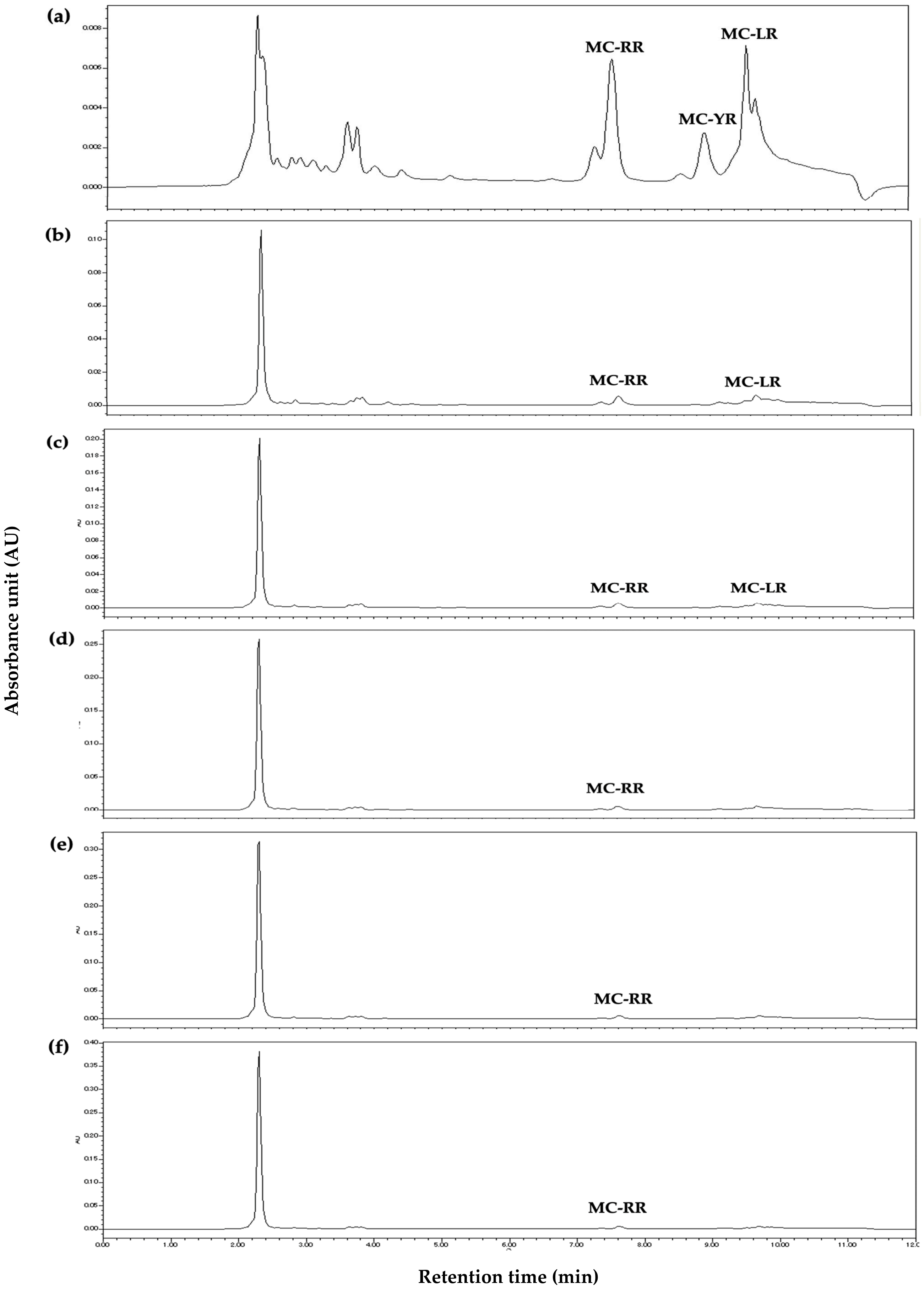

Figure S4. Chromatograms of UPLC analysis of for MC degradation at different temperatures and pH values for strain TA19 at 40 °C and pH 7.0 (a) 0 h, (b) 2 h, (c) 4 h, (d) 6 h, (e) 8 h, (f) 10 h.

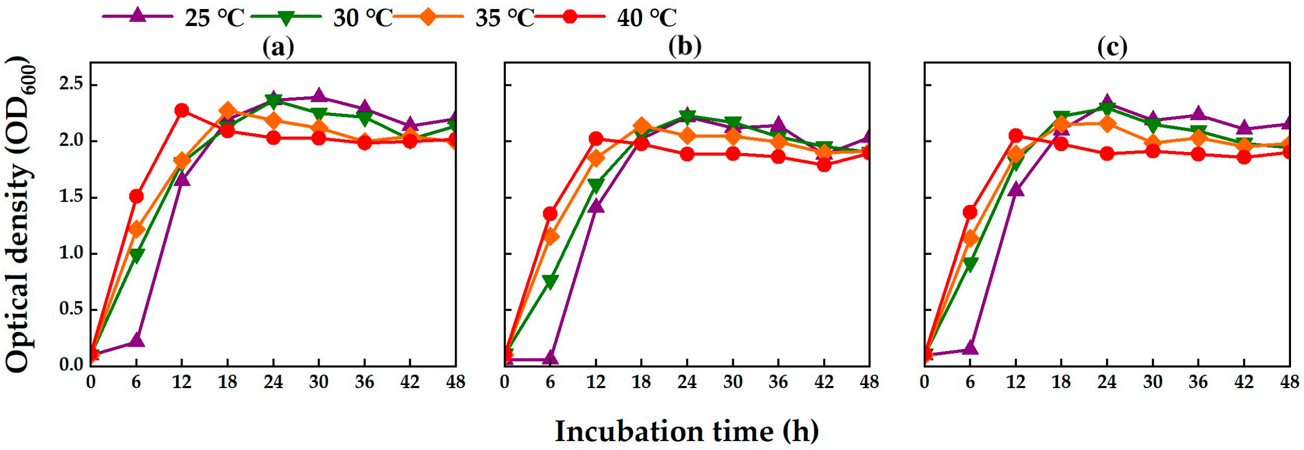

Figure S5. Effect of temperature on growth of the isolated strains (a) TA13, (b) TA14, and (c) TA19.
